# Prognostic Value of sST2 in Heart Failure

**DOI:** 10.3390/jcm12123970

**Published:** 2023-06-11

**Authors:** Edoardo Sciatti, Anna Merlo, Claudio Scangiuzzi, Raul Limonta, Mauro Gori, Emilia D’Elia, Alberto Aimo, Giuseppe Vergaro, Michele Emdin, Michele Senni

**Affiliations:** 1 Cardiology Unit, ASST Papa Giovanni XXIII, 24127 Bergamo, Italy; m.gori@asst-pg23.it (M.G.); e.delia@asst-pg23.it (E.D.); msenni@asst-pg23.it (M.S.); 2 School of Medicine and Surgery, University of Milan-Bicocca, 20126 Milan, Italy; a.merlo@campus.unimib.it (A.M.); c.scangiuzzi@campus.unimib.it (C.S.); r.limonta6@campus.unimib.it (R.L.); 3 Health Science Interdisciplinary Center, Scuola Superiore Sant’Anna, 56127 Pisa, Italy; a.aimo@santannapisa.it (A.A.); vergaro@ftgm.it (G.V.); memdin@ftgm.it (M.E.); 4 Fondazione Toscana G. Monasterio, 56124 Pisa, Italy

**Keywords:** ST2, heart failure, biomarker, natriuretic peptide, prognosis

## Abstract

In recent years, there has been growing interest in the risk stratification for heart failure, and the use of multiple biomarkers to identify different pathophysiological processes associated with this condition. One such biomarker is soluble suppression of tumorigenicity-2 (sST2), which has shown some potential for integration into clinical practice. sST2 is produced by both cardiac fibroblasts and cardiomyocytes in response to myocardial stress. Other sources of sST2 are endothelial cells of the aorta and coronary arteries and immune cells such as T cells. Indeed, ST2 is also associated with inflammatory and immune processes. We aimed at reviewing the prognostic value of sST2 in both chronic and acute heart failure. In this setting, we also provide a flowchart about its potential use in clinical practice.

## 1. Introduction

Heart failure (HF) is a complex clinical syndrome resulting from a diverse range of etiologies. The prevalence of HF appears to be 1–2% of adults, reaching more than 10% of those aged 70 years or more [1]. The prevalence and incidence of HF have been increasing in the last decades due to both better survival of many cardiac diseases and improved care of those patients already diagnosed with HF. Additionally, population aging and the emerging pandemic of cardiovascular (CV) disease in developing countries presage a rise in the incidence and prevalence of HF globally [2]. Nevertheless, mortality and morbidity associated with HF remain high. Recent European data show that the rate of all-cause mortality in 1 year is 8.1% and the rate of hospitalization in 1 year is 28.2% [3]. In this scenario, early diagnosis, accurate identification of disease severity, and risk stratification appear to be crucial to proper HF management.

The most commonly used HF biomarkers in clinical practice are B-type natriuretic peptides (NPs), which are useful in the diagnostic work-up, for risk stratification and in defining the best clinical management. NPs have well-known limitations, as their circulating levels are affected by renal dysfunction, age, obesity, atrial fibrillation, and several cardiac and non-cardiac conditions other than HF [4]. Moreover, although they are considered to be the gold-standard tests to diagnose HF in patients with acute dyspnea, their prognostic utility in the acute setting seems limited [5,6], and their role in guiding treatment has not yet been clearly established [7]. Indeed, the Guiding Evidence-based Therapy Using Biomarker Intensified Treatment in Heart Failure (GUIDE-IT) trial showed that a therapeutic strategy guided by N-terminal pro-B-type NP (NT-proBNP) does not lead to better outcomes than a usual care strategy [8]. In recent years, there has been growing interest in the risk stratification for HF, and the use of multiple biomarkers to identify different pathophysiological processes associated with this condition [9]. One such biomarker is soluble suppression of tumorigenicity-2 (sST2), which has shown some potential for integration into clinical practice [10]. The Serial sST2 Testing in Chronic Heart Failure (STRONG) study demonstrated that serial dosing of sST2, in addition to NT-proBNP, could play a role in optimizing guideline-directed medical therapy (GDMT) for chronic HF patients. This study showed that renin–angiotensin system inhibitors and beta-blockers were associated with significantly lower values of this biomarker, especially when the latter two were introduced and up-titrated [11]. Contrary to the GUIDE-IT trial, these findings suggest that serial sST2 dosing could be useful to optimize GDMT for HF.

More recently, a sub-analysis of the Prospective Comparison of ARNI With Inhibitor to Determine Impact on Global Mortality and Morbidity in Heart Failure (PIONEER-HF) trial demonstrated that sacubitril/valsartan was more effective than enalapril in reducing the aforementioned cardiac stress biomarkers in patients with acute decompensated HF who achieved hemodynamic stabilization, and this reduction was associated with an overall better prognosis [12]. Biomarkers such as NT-proBNP and sST2 could potentially be used as surrogates for clinical outcomes in patients with HF and may be useful in monitoring disease progression and assessing the response to therapy [13].

## 2. ST2 Biology

**Figure 1:**
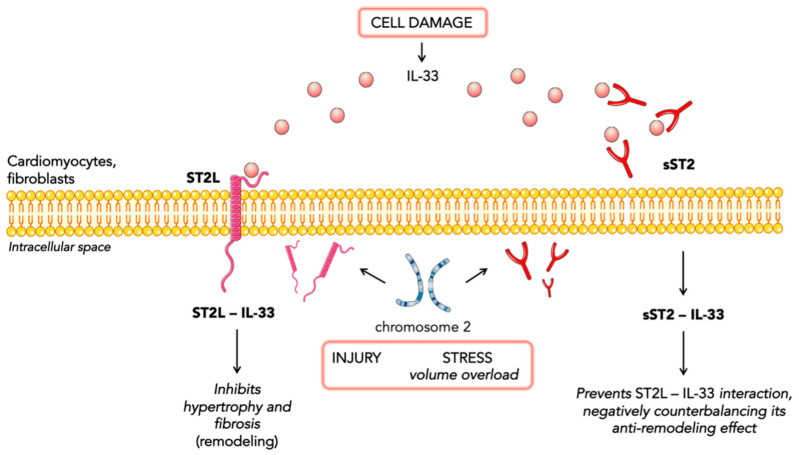
Representation of IL-33 signaling.

ST2 is a member of the interleukin-1 receptor family. The ST2 gene is placed on chromosome 2 and is part of the larger *IL1 gene cluster* [14]. Alternative promoter splicing and 3′-terminal processing of the same mRNA appear to be involved in the generation of two main isoforms: cellular (ST2 ligand or ST2L) and soluble or circulating (sST2) forms [15]. sST2 is a truncated soluble receptor that lacks the transmembrane and cytoplasmatic domains. ST2 is the receptor of IL-33, which is a cytokine secreted by living cells in response to cellular injury or necrosis. IL-33/ST2L signaling is a mechanically activated cardioprotective fibroblast–cardiomyocyte paracrine system, which seems to beneficially regulate the myocardial response to overload and injury [16,17]. Indeed, the interaction of IL-33 and ST2L prevents fibrosis and cardiomyocyte hypertrophy, reduces cellular apoptosis, and, ultimately, improves cardiac function. Such cardioprotective action occurs exclusively through the ST2L receptor and not through the soluble receptor [18]. The circulating isoform (sST2) acts as a decoy receptor and, by sequestrating IL-33, antagonizes the cardioprotective effects of IL-33/ST2L interactions. Both cardiac fibroblasts and cardiomyocytes release sST2 in response to myocardial stress [16,17,19]. Other sources of sST2 are endothelial cells of the aorta and coronary arteries and immune cells such as T cells [20]. Indeed, ST2 is also associated with inflammatory and immune processes, especially regarding the regulation of mast cells and type 2 CD4 T-helper cells [18] (Figure 1).

## 3. Prognostic Value of sST2 in Chronic Heart Failure

**Figure 2:**
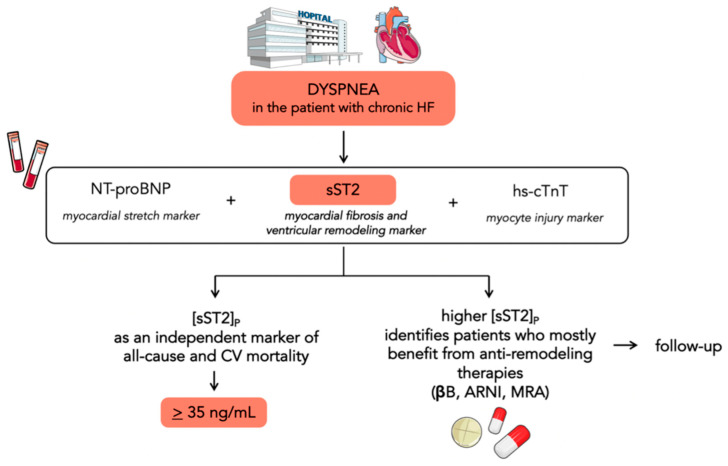
Flowchart showing the potential use of sST2 in chronic heart failure. sST2 is a biomarker of myocardial fibrosis and ventricular remodeling. It could be used in the risk stratification process in addition to NPs and hs-cTnT, as they reflect diverse biologic pathways in HF. Serial testing of sST2 could be helpful in identifying patients who most benefit from anti-remodeling therapies.

sST2 was first evaluated as a HF biomarker in the Prospective Randomized Amlodipine Survival Evaluation 2 (PRAISE-2) trial. Changes in sST2, rather than the baseline value, were a significant independent predictor of mortality or cardiac transplantation in patients with severe HF [21]. The Penn Heart Failure Study on 1141 patients with chronic HF demonstrated that sST2 is a powerful indicator of prognosis and offers a moderate improvement in risk stratification when used in combination with conventional markers (BNP and pro-atrial natriuretic peptide). Higher sST2 levels were associated with a significantly increased risk of all-cause mortality or cardiac transplantation, and this risk was more pronounced in patients with nonischemic HF [22]. The utility of a panel of biomarkers reflecting diverse biologic pathways in HF was further investigated in the Barcelona Study. The authors examined the value of combining NT-proBNP (a marker of myocardial stretch), high-sensitivity cardiac troponin T (hs-cTnT) (a marker of myocyte injury), and sST2 (reflective of myocardial fibrosis and remodeling) (Figure 2). The combined addition of sST2 and hs-cTnT to the model with established risk factors showed a reclassification index of 14%. These findings suggest that the pathways identified by sST2 and hs-cTnT profoundly affect mortality in the context of chronic HF, whereas the information provided in their presence by NPs might be redundant [23]. Accordingly, Emdin and colleagues demonstrated that sST2 yielded a strong predictive value for all-cause and cardiovascular mortality and HF, as well as also improving risk reclassification over NT-proBNP and hs-TnT [24]. Notably, the prognostic value of sST2 was independent of the estimated glomerular filtration rate (eGFR). The inclusion of sST2 along with other biomarkers improved the prediction in patients with renal failure even more than in the global sample population [25]. Gruson and colleagues demonstrated the prognostic value of sST2 for cardiovascular (CV) death over a mean 4.2-year follow-up. sST2 was the strongest predictor of CV death among NPs, age, left ventricular ejection fraction, and eGFR [26]. Galectin-3 (Gal-3) is another biomarker reflective of myocardial fibrosis and remodeling. Higher Gal-3 circulating levels have been associated with the presence of myocardial fibrosis assessed by late gadolinium enhancement (LGE) at cardiac magnetic resonance imaging in nonischemic dilated cardiomyopathy [27]. A cohort study on 876 patients directly compared the two biomarkers of fibrosis (sST2 and Gal-3) and sST2 resulted to be superior over Gal-3 in risk stratification. In addition, both sST2 and Gal-3 were associated with an increased risk of all-cause mortality, but only sST2 with CV mortality. This could be due to the prominent role of Gal-3 in an earlier stage of fibrosis pathobiology and ventricular remodeling, whilst sST2 measurement provides a strong biohumoral overview of the cumulative myocardial fibrotic process [28]. Ky and colleagues identified a cut-off of 36 ng/mL to discriminate patients with chronic HF having a particularly high risk for all-cause death [22]: this cut-off was subsequently confirmed in a post hoc analysis of the PROTECT study [7]. Again, in the PROTECT study, sST2 levels identified patients who may benefit more from higher beta-blocker doses, exploring for the first time the possible interplay between anti-remodeling therapies and sST2 values [29]. The Co-ordinating Study Evaluating Outcomes of Advising and Counseling in Heart Failure (COACH) showed that spironolactone treatment was significantly beneficial in groups with elevated sST2 [30]. The usefulness of serial monitoring of sST2 values over 12 months was evaluated in the Valsartan Heart Failure Trial (Val-HeFT). Increases in sST2 from baseline to 12 months were associated with an increased subsequent risk of poor outcomes and may be useful for monitoring patients. Treatment with valsartan significantly reduced the upward trend in sST2 levels seen in the placebo group [31]. A sub-analysis of the Prospective Comparison of ARNI with ACEI to Determine Impact on Global Mortality and Morbidity in Heart Failure (PARADIGM-HF) showed that sacubitril/valsartan significantly decreased many profibrotic biomarkers and changes from baseline to 8 months in sST2 alongside tissue inhibitor matrix metalloproteinase 1 (TIMP-1) were associated with a change in outcomes [32]. A systematic review of 11 studies with a total of 5121 participants confirmed that higher concentrations of sST2 predict long-term endpoints, such as all-cause mortality, CV mortality or HF-related hospitalization, and all-cause mortality or HF-related readmissions [33]. Furthermore, Vergaro and colleagues analyzed sex-related differences in heart failure biomarkers levels and showed that the optimal sST2 cut-off was approximately 10% lower in women than men. Therefore, risk prediction should consider gender-specific prognostic cut-offs [34].

Lupón et al. developed a score that predicts left ventricular reverse remodeling (RR), named the ST2-R2 score. The variables included in this score were sST2 < 48 ng/mL (3 points), non-ischaemic etiology (5 points), absence of left bundle branch block (4 points), HF duration < 12 months (2 points), beta-blocker treatment (2 points), and baseline left ventricular ejection fraction (LVEF) < 24% (1 point). The frequency of RR ranged from 10% in patients with scores of 2–5 to 86% in patients with scores of 15–17. The score had an area under the curve value of 0.79 in the derivation cohort and 0.73 in the validation cohort. Furthermore, there was a gradual increase in LVEF (from +5.6% to +17.3%; *p* < 0.001), and a progressive reduction of LV end-systolic volume index (from −6.1% to −32.1%; *p* < 0.001) and in LV end-systolic diameter index (from −1.1% to −18.6%; *p* < 0.001) across ST2-R2 score values [35]. The ST2-R2 score was also shown to be predictive of all-cause mortality for up to 4 years [36].

Interestingly, it has been shown that sST2 level is an independent influencing factor associated with atrial fibrillation (AF) in HF patients [37], possibly reflecting both aging and remodeling phenomena that affect the atrium in AF patients [38]. Moreover, elevated sST2 has been associated with a higher risk of recurrence of AF after radiofrequency catheter ablation (RFA); hence, sST2 concentration might be used as a pre-diagnostic marker of FA recurrence [39].

Despite this growing body of evidence, while the 2013 clinical practice guidelines from the American College of Cardiology/American Heart Association have given a Class IIb recommendation for the sST2 measurements in chronic HF for the purpose of risk stratification and prognostication [40], the 2022 guidelines only refer to the use of NPs as biomarkers [41], similarly to the European Society of Cardiology guidelines [1].

## 4. Prognostic Value of sST2 in Acute Heart Failure

sST2 is a promising biomarker of acute HF (AHF), being released following hemodynamic congestion as well as cell damage and inflammatory activation. By reflecting different pathophysiologic pathways, biomarkers such as sST2, mid-regional proadrenomedullin (MR-proADM), NPs, and C reactive protein (CRP) may convey additive information on the individual pathophysiology and final outcomes.

Patients with stable HF usually have low sST2 levels, whereas in AHF the increase in plasma levels provides valuable prognostic information [42,43]. Aimo and colleagues demonstrated that concentration of sST2 was predictive of all-cause death, CV death, and the composite outcome of all-cause death or HF hospitalization in AHF patients. Importantly, not only admission values were prognostic, but also discharge values predicted HF re-hospitalization [43].

In the Translational Initiative on Unique and novel strategies for Management of Patients with Heart Failure (TRIUMPH) clinical cohort study, 496 patients with acute HF were enrolled in 14 hospitals in the Netherlands between 2009 and 2014, and repeated blood samples were drawn during 1-year follow-up. The primary endpoint of the study was the composite of all-cause death mortality and readmission for HF. Results from the van Vark et al. study showed that, in the highest quartile of baseline sST2, 52% of the patients reached the primary endpoint compared with 23% in the lowest quartile of sST2. Hence, this study demonstrates that baseline sST2 levels, and especially repeated sST2 measurements, are strong and independent predictors of adverse outcomes in patients following admission for acute HF [44].

It appears that, in AHF, hemodynamic congestion and inflammation activate vascular endothelial cells and lung tissue that release pro-inflammatory cytokines, which are responsible for the upregulation of sST2 [45,46,47]. sST2 may be a valid surrogate biomarker of systemic and pulmonary congestion in HF because it correlates positively with both echocardiographic indicators of right-sided HF and invasively measured central venous pressure [48,49,50].

A post hoc analysis of the Registry to Improve the Use of Evidence-based Heart Failure Therapies in the Outpatient Setting (IMPROVE HF) trial investigated whether the circulating levels of sST2 could predict the cumulative diuretic efficiency in patients with AHF and renal dysfunction at presentation. It seems that circulating levels of sST2 are independently and negatively associated with a poor diuretic response [51]. Further studies are needed to evaluate the aforementioned association since early detection of patients at risk of poor diuretic response could be helpful to identify those who are in need of more aggressive treatment.

The pro-BNP Investigation of Dyspnea in the Emergency Department (PRIDE) study was a prospective, blinded study carried out on 599 dyspneic subjects who attended the ED of the Massachusetts General Hospital. The main purpose of the study was to validate the use of NT-proBNP testing; moreover, blood collected at the time of presentation was also analyzed for concentrations of ST2 and other markers. Patients were divided into ST2 deciles, and the frequency of mortality relative to increasing ST2 concentrations above or below the median was estimated with odds ratios (OR) with 95% confidence intervals (CI). Elevated sST2 concentrations strongly predicted death at 1 year in dyspneic patients (HR 5.6; 95% CI, 2.2–14.2; *p* < 0.001), as well as in those with acute decompensated HF (ADHF) (HR 9.3; 95% CI, 1.3–17.8; *p* = 0.03) above and beyond NT-proBNP. Patients with the highest risk of death could be more accurately identified by a multimarker approach that included sST2 and NT-proBNP. The ability of sST2 to predict risk was similar in those with HFpEF and HFrEF (HFpEF, per ng/mL, HR 1.41, 95% CI, 1.14–1.76, *p* = 0.002; and HFrEF, per ng/mL, HR 1.20, 95% CI, 1.10–1.32, *p* < 0.001).

**Figure 3:**
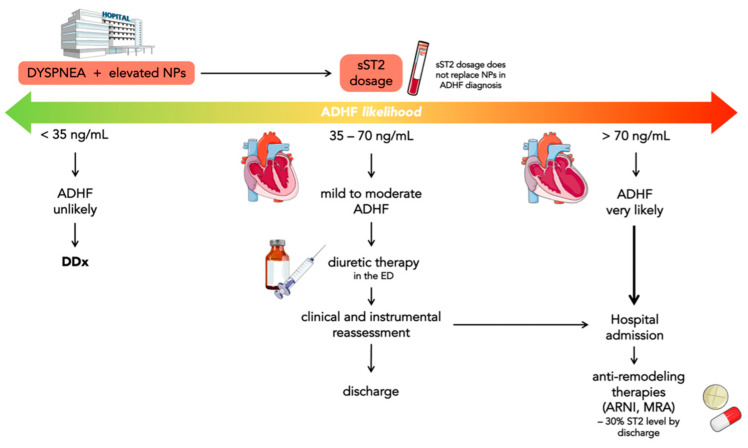
The proposed sST2-aided management of patients with dyspnea and elevated NPs is shown in the flowchart. In dyspneic patients with elevated NPs, sST2 levels can help to identify 3 classes of patients. If sST2 < 35 ng/mL, the diagnosis of acute decompensated heart failure (ADHF) is unlikely. In patients with 35 ≤ sST2 ≤ 70 ng/mL, ADHF is more common but mild to moderate. If sST2 > 70 ng/mL, ADHF is common, requiring hospitalization and anti-remodeling therapies. The panels below suggest actions for each class of patients.

Januzzi et al. provided a flowchart about sST2 use to differentiate acute dyspnea (Figure 3). A cut-off value of sST2 ≥ 35 ng/mL identified patients with acute HF and worse prognosis [52]. It is possible to divide all patients with dyspnea and high NPs levels into three groups based on the level of sST2:

- sST2 levels <35 ng/mL have been found in less than 10% of ADHF in ED; consequently, ADHF can be reasonably ruled out in these patients;
- sST2 levels between 35 and 70 ng/mL may suggest evaluating the outcome of diuretics efficacy directly in ED and, if there are improvements in symptoms, establish whether patients need hospitalization or not;
- sST2 levels >70 ng/mL indicate patients with a very high risk of ADHF who need hospitalization.

However, using a prognostic tool as a diagnostic one could be a problem. In fact, the use of sST2 as a diagnostic tool has not been validated yet.

Indeed, sST2 values above approximately 70 ng/mL have been associated with a higher risk of death on both a short- (30 days) and long-term (one year) follow-up [53]. The reason could be the significant activation of the neurohormonal and fibrotic pathways, which induce adverse myocardial remodeling after an acute event. The above suggests that in patients presenting in the ED with dyspnea and elevated NPs, an sST2-based flowchart could be useful for a more accurate diagnosis, risk stratification, and treatment [54].

A cohort of 150 patients, suffering from decompensated HF, had their blood drawn daily and ST2 measured. During the observation period, the group of patients who had a 16% or greater ST2 values decrease had a 7% mortality risk, while the group that did not have a 16% decrease in ST2 values had a mortality risk of 33%. Thus, this study demonstrated that the percentage change in ST2 was highly predictive of 90-day mortality and the prognostic value of sST2 changes was independent of variations in NT-proBNP [55]. In a sub-analysis of the PIONEER-HF trial (comparison of the effect of sacubitril/valsartan vs. enalapril on NT-proBNP in patients stabilized after an AHF episode), a significant reduction of circulating sST2 values as early as 1 week was demonstrated in patients in the sacubitril/valsartan arm compared to patients treated with enalapril. Baseline sST2 concentrations have been shown to have prognostic significance for the composite outcome of cardiovascular death or HF rehospitalization [12].

Finally, in the context of acute coronary syndromes, both baseline and serial measurements of ST2 can accurately predict future cardiovascular events [56]. In particular, increased serum levels of sST2 one week after a ST segment elevation myocardial infarction were associated with the development of adverse left ventricular remodeling at 6 months [57].

## 5. Conclusions

sST2 provides important information about prognosis in HF and it is less influenced by renal function, age, body mass index, and etiology than NPs. Although still not widespread, it is able to be measured easily and repeatedly in emergency situations and daily clinical situations. In addition, it is stable for days after sampling. Hence, future studies should focus on optimizing the use of sST2, including examining its role to inform therapeutic decision-making in CHF as in ADHF. More studies are needed to define the optimal cut-offs and to demonstrate if “new drugs”, such as SGLT2i or vericiguat, could have effects on sST2 reliability. Using more than one tool can help clinicians to better assess patients’ outcomes by identifying those patients who need hospitalization and a more intensive level of care, given that high sST2 levels are an indicator of higher cardiac remodeling and thus a more compromised situation. Consequently, adopting the proposed flowchart and dosing sST2 may be useful in clinical practice.

## Data Availability

Not applicable.
